# Exploring facilitators and barriers of the sustainable acceptance of e-health system solutions in Ethiopia: A systematic review

**DOI:** 10.1371/journal.pone.0287991

**Published:** 2023-08-10

**Authors:** Agmasie Damtew Walle, Addisalem Workie Demsash, Jibril Bashir Adem, Sisay Maru Wubante, Adamu Ambachew Shibabaw, Daniel Niguse Mamo, Shimels Derso Kebede, Ayana Alebachew Muluneh, Muluken Belachew Mengiste, Ayenew Sisay Gebeyew, Fikadu Wake Butta, Alex Ayenew Chereka, Abiy Tasew Dubale, Sisay Yitayih Kassie, Tigist Andargie Ferede

**Affiliations:** 1 Department of Health Informatics, College of Health Science, Mattu University, Metu, Ethiopia; 2 Department of Public Health, College of Medicine and Health Sciences, Arsi University, Asella, Ethiopia; 3 Department of Health Informatics, Institute of Public Health, College of Medicine and Health Sciences, University of Gondar, Gondar, Ethiopia; 4 Department of Health Informatics, College of Medicine and Health Sciences, Arba Minch University, Arba Minch, Ethiopia; 5 Department of Health Informatics, School of Public Health, College of Medicine and Health Sciences, Wollo University, Dessie, Ethiopia; 6 Department of Health Informatics, College of Health Sciences, Debre Markos University, Debre Markos, Ethiopia; 7 Department of Epidemiology and Biostatistics, Institute of Public Health, College of Medicine and Health Sciences, University of Gondar, Gondar, Ethiopia; Universiti Tenaga Nasional, MALAYSIA

## Abstract

**Background:**

eHealth is the use of information and communications technologies in support of health and health-related fields, including healthcare services, health surveillance, health literature, and health education knowledge and research, has the potential to improve the delivery and support of healthcare services by promoting information sharing and evidence-based health practice. Acceptance of e-health in Ethiopia using systematic review is uncertain. As a result, this study aimed to assess barriers and facilitators of the sustainable acceptance of e-health system adoption in Ethiopia through a systematic review of the literature.

**Methods:**

The Preferred Reporting Items for Systematic Reviews and Meta-Analysis (PRISMA) checklist was used to conduct this study. Relevant articles have been searched in Google Scholar, Medline, PubMed, Embrace, Web of Science, Scopus, Cochrane Library, and empirical research done in Ethiopia is the main emphasis of the search strategy. The total number of studies that satisfied the criteria for inclusion was ten. In this research, empirical data related to e-health acceptance factors were retrieved, examined, and summarized by the authors.

**Results:**

This systematic review identified a total of 25 predictors that have been found in the ten studies. The identified facilitators were effort expectancy, performance expectancy, facilitating conditions, social influences, attitude, computer literacy, participant age, perceived enjoyment, and educational status, duration of mobile device use, organizational culture, and habit. Moreover, technology anxiety was the most barrier to sustainable acceptance of e-health systems in Ethiopia.

**Conclusions:**

The most common facilitator identified from the predictors was effort expectancy, which played a major role in the adoption of the e-health system in Ethiopia. Therefore, eHealth implementers and managers in those settings should give users of the system priority in improving the technical infrastructure by regularly providing them with basic facilitating conditions. They should also pay attention to the system they want to implement because doing so will improve the users’ perception of the system’s value and attitude.

## Introduction

Poor healthcare systems increase morbidity and mortality rates, which hinder economic growth and profitability in nations [1]. Thus, the delivery and quality of services in healthcare systems can be greatly improved by the use of digital health solutions like eHealth. eHealth, which is defined as the use of information and communications technologies (ICT) in support of health and health-related fields, including healthcare services, health surveillance, health literature, and health education knowledge and research, has the potential to improve the delivery and support of healthcare services by promoting information sharing and evidence-based health practice [2, 3]. Health services and information offered or improved through the internet and other associated technologies are referred to as being provided through eHealth, an emerging sector at the intersection of medical informatics, public health, and business [1, 4]. Moreover, improving the healthcare system requires sustainable acceptance of eHealth, which is the process of a shift in both thinking and practice of the benefit when it comes to the spread and adoption of healthcare technologies [5].

Hospital information systems (HIS), electronic health records (EHR), mobile health (M-health), decision support systems (DSS), electronic medical record systems (EMR), data collection systems (RCS), and Telemedicine services are solutions for health technology applications that have significant value in various healthcare settings by lowering healthcare costs, advancing access to current information, enabling quick access to patient records, and improving communication between patients and healthcare professionals [6–8]. The practical application and integration of eHealth systems in Europe have grown significantly during the last ten years [9]. The use of eHealth technology in many industrialized countries has a significant impact on patient care and the delivery of effective healthcare services [9, 10].

The pressure on healthcare spending may be seen in the case of the Netherlands, where without action, healthcare spending would increase from 15.6% of GDP in 2013 to 22–31% of the nation’s GDP in 2040. Additionally, to meet the need for healthcare in 2040, the healthcare sector will need to employ 25% of the working population [4, 11, 12]. Policymakers are promoting the potential of eHealth in sustaining the healthcare system at both the national and European levels [4, 13]. Additionally, there is a significant divide between European nations, with the Nordic nations; Denmark, Estonia, Sweden, and Finland, performing the best while other Eastern European nations and Greece exhibit less sophisticated usage of e-health [4]. However, in any nation, introducing and integrating technological advancements of any kind necessitates complicated processes of learning and development at the meso-level of healthcare organizations as well as the micro-level of medical professionals and patients [14].

eHealth has attracted a lot of attention, but the adoption and acceptance rates have not been high enough for healthcare systems to realize all of its advantages [10]. The practical application and integration of eHealth systems have grown significantly during the last decade and by utilizing eHealth technologies, several industrialized nations are significantly enhancing patient care and the provision of cost-effective healthcare services [3]. Although the progress and success rate in the underdeveloped world is not pleased, there are trials and deployments of eHealth technology in various areas of the healthcare industry [15]. Inappropriate changes in documentation procedures, patient alerts, teleconsultation, and evidence-based practice were evident, and the government is placing more focus on system deployment as the need to identify key elements for the long-term uptake of eHealth technology rises [16].

Several eHealth technologies, including Smart Care, mobile ENAT Messenger, maternal Interactive Voice Record (IVR), Health Management Information System (HMIS), EMRs (Electronic Medical Record System), DHIS-2 (District Health Information System version 2) have been introduced in Ethiopia [3]. Concerning this performance expectancy, effort expectancy, social influence, attitude, perceived enjoyment, trust, facilitating conditions (availability of technologies), internet access, habit, and educational level was important to improve e-health solution [7, 17, 18].

All currently deployed systems and those that have been deployed in the past have all been implemented through a very costly and ineffective process, moreover, a lack of access to the required electronic equipment (i.e., smartphone, tablet, or computer) and lack of technological support (e.g., training, troubleshooting, and guidance) provided alongside e-health programs was a barrier to uptake, unreliable or unavailable internet services, lack of social interaction, privacy, and security, mistrust of e-health and lack of information communication between health platforms and professionals considered as barriers of acceptance e-health solutions [8, 19]. As a result, eHealth technology adoption and dissemination in Ethiopia are still in their infancy [19–21].

There is no doubting the growth of information technologies in the healthcare industry [22]. eHealth technologies are crucial to raising the standard of healthcare and increasing patient happiness. Also, the use of technology by healthcare professionals is a crucial problem because information technologies are key to improving their productivity and effectiveness at work [23]. Many theoretical models and their expansions have been developed over the past three decades to comprehend the levels of acceptability and people’s actions toward various technology in various fields [18, 23]. The UTAUT is regarded as the most pertinent and frequently utilized model among the aforementioned theories and models in studies of technology adoption in the healthcare industry [24, 25]. TAM is also considered the gold standard model across several technologies, aside from the healthcare domain [24].

On the other hand, UTAUT has demonstrated 20–30% stronger explanatory power than TAM, which equates to 40–50% of explanatory power regarding the behavioral intention of end-users [26, 27]. This is why it’s critical to ascertain and comprehend how people respond to the advancements in health technology. Implementing a certain information technology could be unsuccessful or take longer due to low levels of adoption. Furthermore, the failure to adopt technology in healthcare can affect the healthcare systems [24]. Accordingly, understanding the facilitators of sustainable acceptance and use of eHealth technology in the resource-limited setting is very critical. Additionally, developing nations received less attention as eHealth solutions were mostly examined from the perspective of elderly people [28].

The study made the following contributions to the literature. It is one of the first of its kind to provide a more thorough image and understanding of eHealth adoption in hospitals by differentiating numerous stages from earlier studies, including a more sophisticated design and early testing of various adoption-related components. The study also applies the TOE framework (technological, organizational, and environmental) to a hospital setting, with a focus on the organizational context. Although studies are being conducted to identify the facilitators of the acceptance and adoption of eHealth services in Ethiopia, there hasn’t been a thorough, systematic analysis of those studies. Thus, our study aimed to identify barriers and facilitators of the sustainable acceptance of e-health system adoption in Ethiopia, using a systematic review of the literature.

Finally, this study’s address the following research questions:

1. What is the primary technology acceptance model in the study to determine the significant predictors of eHealth systems acceptance?
2. What are the key facilitators of eHealth system acceptance in the healthcare domain?
3. What are the barriers to influencing sustainable acceptance of eHealth systems in Ethiopia?

## Materials and methods

### Source of information and search strategy

The Preferred Reporting Items for Systematic Reviews and Meta-Analysis (PRISMA) checklist was used to conduct the research, to identify the factors that facilitate the adoption of e-health systems (S1 Table).

A review strategy was created by the study team. A database search was conducted online between September 5 and January 10 of 2022. We searched the databases Google Scholar, Medline, PubMed, Embrace, Web of Science, Scopus, Cochrane Library, and another online database to find relevant published works until January 10, 2023. Medical Subject Headings (Mesh), keywords, and free text search queries were all used as search terms. We incorporate alternative words and combine them using Boolean operators. We used the following search terms: ((“eHealth” OR “e-health” OR “telemedicine” OR “telehealth” OR “telecare” OR “remote health” OR “mhealth” OR “mobile health” OR “EMR” OR “electronic medical record” OR “HER” OR “electronic health record”) AND (“adoption” OR “intention” OR “acceptance”) AND (“Ethiopia”)). The protocol number for this systematic review is CRD42023399513 in Prospero.

### Eligibility criteria

The study contains original research papers from Ethiopia that examined the factors influencing people’s acceptability of using e-health services. This study covered studies that were openly available in full-text, English-language publications from peer-reviewed journals or publications between the years 2010 and 2023. Studies that lacked comprehensive texts had data that was difficult to extract, weren’t published in English, or didn’t address participants’ acceptance or desire to use e-health systems in Ethiopia were excluded.

### Data extraction and analysis

The data from the included studies were compiled into a Microsoft Excel spreadsheet by three authors, and the accuracy was checked by the remaining researchers. For each study, the initial author’s name, the year it was published, types of research, sample size, sampling technique, study population, data collection techniques, and the study’s design were all gathered. Parameters were also retrieved, and discrepancies amongst data extractors have been resolved through discussion with the authors, via the PRISMA standard. Moreover, the article is moved to the "Resolve disputes" list if the authors cannot agree on whether or not to include the study or the justification provided for its exclusion.

The reviewers must once more agree. Again, if they are unable to resolve the issue, it is forwarded to another team member, with the final decision. As they read through the references, reviewers are unaware of other people’s votes. This will prevent the decisions they make from being influenced by those of their colleagues. Upon the completion of the voting process and the resolution of any conflicts, the authors simply generate a flow diagram using the decision data. This follows the reporting criteria and records the progression of the studies through the review. Hence to resolve conflicts, blinding was turned on (which can only be done by the creator of the review). Furthermore, study the flow of studies through the review follows the reporting standards for systematic reviews as set out in the PRISMA statement (S1 Table).

In this study, a meta-analysis of the finding was not conducted due to differences in methods and participant types between studies. For instance, this study used both quantitative and qualitative methods to investigate the elements that make acceptance of e-health technology. The study’s participants also varied (healthcare workers, patients, and students). Moreover, some studies’ statistical interpretations that supported the conclusions were not practicable. Accordingly, the current study adopted the analytical approach used in earlier systematic reviews. In this way, the facilitators were examined based on how frequently they appeared in the studies and the investigation of this approach resulted in accurate findings.

### Quality assessment and appraisal

The quality of each study was assessed using a standardized tool that classifies bias potential and can be used to explain discrepancies in the findings of included studies [7]. Three writers independently performed a quality control check. In addition, the authors assessed the methodological and other elements of each publication using a modified version of the Newcastle-Ottawa Scale (NOS) for cross-sectional research, a valid instrument for measuring bias risk in observational studies [29, 30]. After analyzing the publications, we concluded that publications scoring seven or higher on the modified NOS components were relevant (S2 Table).

### Patient and public involvement

The research question, outcome measures, design, recruitment, analysis and interpretation of the results and study execution were all developed without the involvement of any patients. Additionally, there are no plans to share the findings with the patients and the study’s design did not directly engage the general public.

### Ethical consideration

Since this study is systematic review or review the existing studies via the Preferred Reporting Items for Systematic Reviews and Meta-Analysis (PRISMA) checklist. We also, registered the review in Prospero with the protocol number of CRD42023399513. Moreover, we frequently read and cited the included studies in the manuscript for addressing the issue of the potential conflicts of interest and issues of voice and representation. As a result, it is not applicable for ethics approval as well as the participant recruitment dates and/or date on which medical records were accessed.

## Results

### Study selection

**Fig 1:**
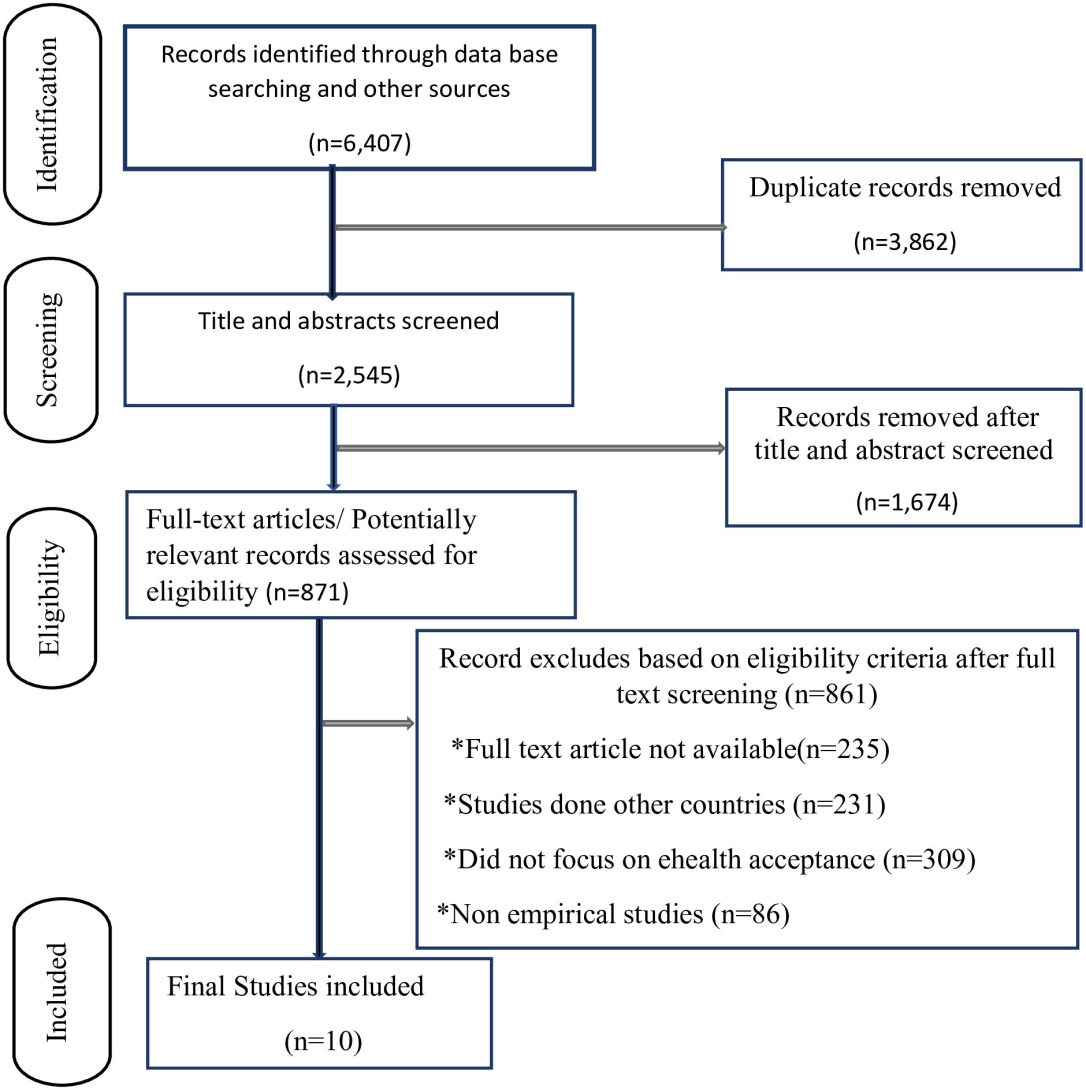
PRISMA flowchart showing the selection process of the articles.

There were 6,407 articles in all that were found. The title and abstract of 2,545 titles were checked after removing 3,862 duplicates, and 1,674 records were eliminated. Following the full-text screening using the inclusion and exclusion criteria, 861 articles were removed. Among those papers, they consider the systematic review. Finally, 10 papers were included in the study based on the pre-established criteria and quality evaluation (Fig 1).

### Characteristics of included studies

In the final systematic review, ten studies from Ethiopia were included. In all eight studies, except one mixed-method [31] and one qualitative study [32], nine cross-sectional quantitative design study was included. Seven of these studies among healthcare professionals, one done on the student [33], one among reproductive healthy women [21], and one among DM patients were conducted [18]. Six of these studies used only a self-administered questionnaire of data collection method, furthermore, four studies used the UTAUT model (S1 Data) (Table 1).

**Table 1:** Characteristics of included studies.

Author, publication year	Types of research	Model used	Study design	Study population	Sample size	Sampling technique	Data collection method	Quality score
Ahmed. et al, 2020 [31]	Mixed	UTAUT2	Cross-sectional	Healthcare professionals	420	Simple random	Questioner & Interview	8
Shiferaw. et al, 2019 [34]	Quantitative	TAM	Cross-sectional	Healthcare professionals	405	Simple random	Questioner	7
Kalayou. et al, 2020 [3]	Quantitative	TAM	Cross-sectional	Healthcare professionals	384	Simple random	Questioner	9
Walle. et al, 2022 [18]	Quantitative	UTAUT2	Cross-sectional	DM Patients	883	Systematic random	Interview	9
Shiferaw. et al, 2021 [35]	Quantitative	UTAUT	Cross-sectional	Healthcare professionals	319	Simple random	Questioner	8
Mekonnen. et al, 2021 [21]	Quantitative	Others	Cross-sectional	Women	456	Simple random	Interview	9
Hunde. et al, 2022 [33]	Quantitative	UTAUT	Cross-sectional	Students	637	Stratified	Questioner	9
Kifle. et al, 2010 [36]	Quantitative	UTAUT	Cross-sectional	Healthcare professionals	144	Survey	Questioner	7
Bramo. et al, 2022 [32]	Qualitative	UTAUT	Ethnographic	Healthcare professionals	38	Purposive	Observation & interview	9
Walle. et.al 2023 [17]	Quantitative	TAM	Cross-sectional	Healthcare professionals	610	Simple random	Questioner	9

### Facilitators and barriers to sustainable acceptance of e-health system in Ethiopia

In this systematic review, a total of 12 facilitators and 13 barriers have been found in the 10 studies. The identified facilitators in the information technology model constructs were effort expectancy, performance expectancy, facilitating conditions, social influences, and attitude, whereas computer literacy, participant age, perceived enjoyment, educational status, duration of mobile device use, organizational culture, and habit. Whereas older age, lack of interest to use, perceived systems are not saving time and money, unable to share knowledge, and not experienced work, unclear platforms of the system and not easy of the systems to do their work, lack of infrastructure, lack truth worthiness of the systems, lack of date health information in an organization, lack of system training, and lack of leadership commitment were barriers of sustainable acceptance of e-health systems in Ethiopia.

**Fig 2:**
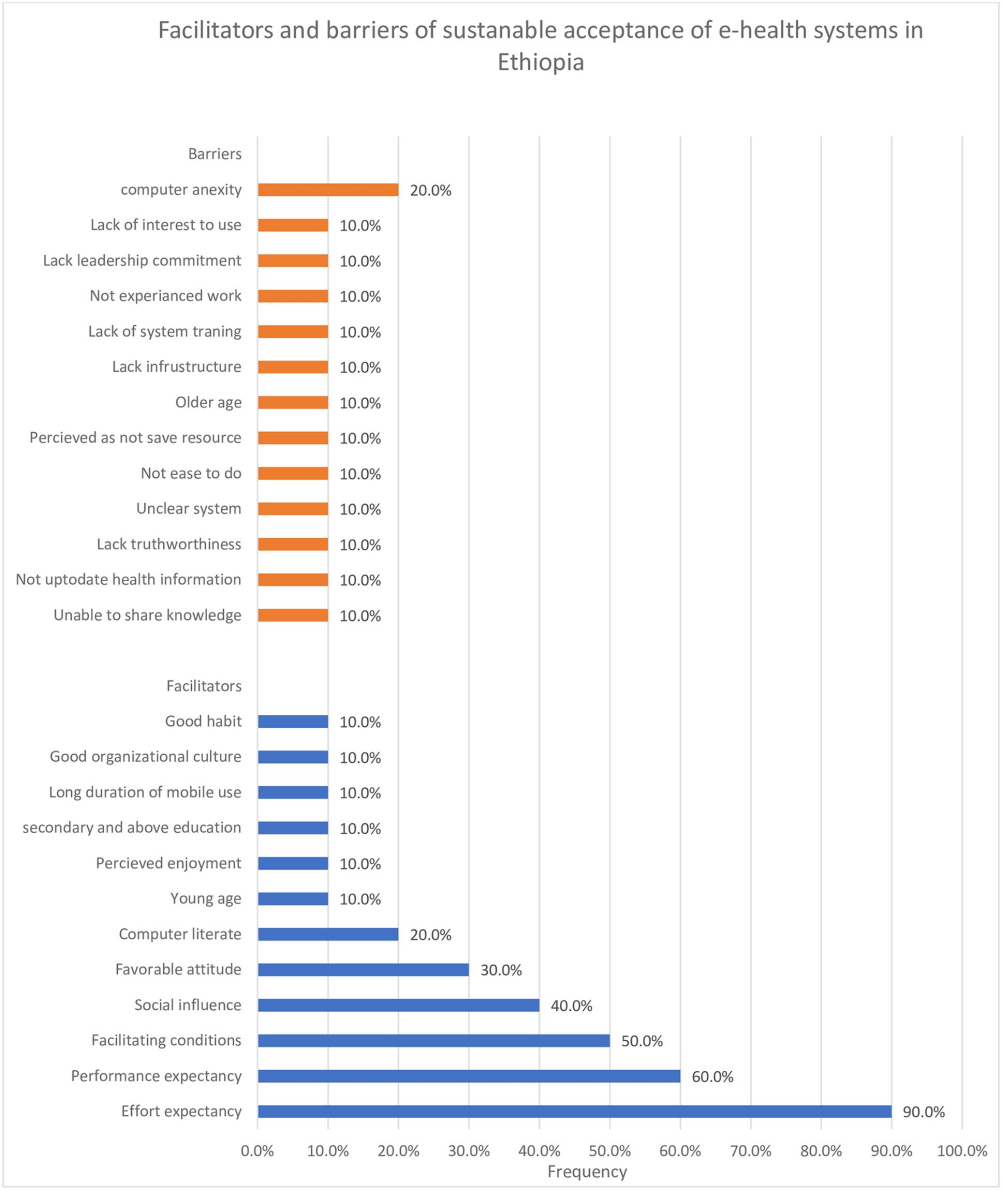
Facilitators and barriers of their percentage occurrence in the sustainable acceptance of e-health system adoption in Ethiopia.

The factors were presented in order of how frequently they appeared in the studies using percentages, with the most frequent ones being listed first. The accessing of barriers and facilitators of sustainable acceptance of e-health was reported systematically into four thematic categories (individual, technological, organizational, and social) [8] (Fig 2).

### Individual factors

The study finding revealed that participants who were grouped young age (10.0%) [21], secondary and above educational level (10.0%) [21], perceived enjoyment (10.0%) [33], favorable attitude (30.0%) [3, 17, 35] and good habit (10.0%) [18] frequently occurred in studies and the individual facilitators of sustainable acceptance of e-health systems in Ethiopia. Whereas anxiety(fear of new technologies) appeared in 2 studies (20.0%) [8, 32]. In addition, older age, lack of interest to use, perceived as systems are not save time and money, unable to share knowledge, and had not experienced work were the individual barriers to sustainable acceptance of e-health systems in our study, which occurred once from the study (10%) [32].

### Technological factors

The study showed that the individual who had positive effort expectancy (90.0%) [3, 17, 18, 21, 31–35], performance expectancy (60.0%) [3, 18, 31, 32, 34, 36], computer literacy (20.0%), long duration of mobile devices use (10.0%) [21] were technological facilitators of sustainable acceptance of e-health system in Ethiopia. Whereas, 10% of unclear platforms of the system and not ease of the systems to do their work were technological barriers to sustainable acceptance of e-health system [32].

### Organizational factors

This study showed that participants who had more facilitate conditions (50.0%) [3, 18, 31–33] and good organizational culture (10%) [32] were important facilitators of sustainable acceptance of e-health systems. Whereas, lack of infrastructure, lack truth worthiness of the systems, lack of date health information in an organization, lack of system training, and lack of leadership commitment were significant barriers to sustainable acceptance of e-health systems in resource-limited setting (Ethiopia)with the frequency of occurrence in the study(10%) [32].

### Social related factors

Social influence occurred in four of ten studies (40.0%) [31, 32, 34, 36] and was the best predictor to influence facilitators of sustainable e-health systems in Ethiopia. However, there were no barriers to social-related factors influencing sustainable e-health systems among the included studies.

## Discussion

This systematic review identifies potential health technology models, barriers, and facilitators of the acceptance of e-health systems implementation in Ethiopia by critically reviewing and synthesizing the research that has already been done on e-health acceptance studies using 4 thematic categories (individual, technological, organizational, and social). Moreover, the results of this study will help policymakers plan and create strategies to increase the adoption of e-health in Ethiopia.

The model used to identify facilitators of sustainable acceptance of eHealth in resource-limited settings was the unified theory of acceptance and use of technology, in addition, the most common facilitators (which occurred frequently in the studies) were effort expectancy, performance expectancy, and facilitating conditions, social influences, and attitude. Whereas, fear of new technology (anxiety) was the frequent barrier to influencing sustainable acceptance of the e-health system in Ethiopia.

According to technological facilitators, effort expectancy was the most frequent facilitator of the acceptance of e-health technology across Ethiopia. According to a systematic review of previous studies, effort expectancy or perceived ease of use was a significant factor for the successful implementation of an e-health system [1, 37–45]. This suggested that a person’s perspective and acceptance of using an e-health system will be improved if they think it will be convenient. This may be because the effort required to manage the system has a significant impact on people’s attitudes, perceptions of their usefulness, and intentions to utilize eHealth systems. Health systems will be improved if the system is anticipated to require less effort to control people’s acceptance of using it. For healthcare professionals as well as patients to utilize eHealth technology sustainably in the future, the system needs to be simple for them to use and comprehend [3].

Another technological factor of the facilitators to influence the sustainable acceptance of e-health was performance expectancy or perceived usefulness. This revealed an increase in users’ performance expectation acceptance of using e-health systems also rises. This finding is supported by a systematic review conducted in previous studies [39–46]. This may be because the use of e-health technologies for optimizing daily workflow and improving user safety influences people’s acceptability of the adoption of e-health technology in the work environment [31].

Accordingly, performance expectancy should be considered an important predictor for the successful implementation of e-health technologies in Ethiopia. Moreover, computer literacy is also the facilitator of e-health system acceptability. This could be because those who are computer knowledgeable think, they won’t have any problems with the acceptance of an e-health system adoption [31]. Unclear platforms of the system and not ease of the systems to do their work were technological barriers to sustainable acceptance of e-health system. This could be due to people expressing their dislike of newer technologies that need extensive training to operate. Also, the field observation revealed that people are hesitant to test new technologies in healthcare settings because they are afraid of the unknown, thus the systems need to be user-friendly and simple to use [30, 32].

Facilitating conditions are also important organizational facilitators for the full acceptance of e-health technologies in resource-limited settings. This shows that an increase in users’ facilitating condition leads to an increase in acceptance of using e-health systems. This finding is supported by a systematic review conducted in previous studies [23, 38–43]. It might be because consumers think Ethiopians should have access to the resources and technical support, they need to use e-health services effectively. Healthcare professionals and patients may believe that taking training will improve their ability to use e-health in the future because training and organizational preparation are key components of facilitating conditions.

Moreover, the lack of system training was a significant organizational barrier to sustainable acceptance of e-health systems in resource-limited setting (Ethiopia). This finding was in line with the systematic study of electronic medical record systems adoption in Ethiopia [30, 47]. This result indicates that continued e-health system development and fundamental computer training may have a significant impact on the healthcare system’s adoption of e-health. This suggests that to enhance the degree of e-health use and guarantee its successful implementation, the Ethiopian Ministry of Health should become ready to provide users with comprehensive end-user training packages [30]. Lack of leadership commitment was also a barrier to acceptance of the e-health system. As a result, the way the commitment of leaders of healthcare facilities encourage their staff to be innovative and open-minded in experimenting with new technology and procedures in healthcare facilities has motivated the users [32].

Social influence also played a significant role to facilitate the acceptance of users as well as the implementation of the e-health system in Ethiopia. This result was supported by a systematic review conducted in previous studies [38–43, 45]. This showed that Users may believe that patients, healthcare providers, or hospital administration are pressuring them to use a new system. Customers must therefore feel pressure from an outside source to boost their motivation and acceptance for using e-health systems. Mechanisms that promote peer support, role modeling, and super users may improve Ethiopians’ acceptance of e-health services. Therefore, in areas with limited resources, social influence should be considered a key predictor of the adoption of e-health technologies.

Moreover, the individual facilitator of attitude played a crucial role in facilitating user acceptance and the adoption of the e-health system in Ethiopia. This revealed that when the user’s attitude improves the adoption of e-health technologies in resource-limited settings will be enhanced. This finding was congruent with a systematic review done in previous studies [39, 41–43, 45, 46]. This may be because users who already have a favorable attitude toward eHealth systems will be extremely incensed by new eHealth solutions. Therefore, a strong emphasis should be placed on initiatives that improve attitudes, such as providing computers at work, providing ongoing training and support, and sharing knowledge about eHealth technologies [3].

However, anxiety was the most barrier to influencing the sustainable acceptance of e-health systems in Ethiopia, this could be due to in resource-limited setting persons are the socioeconomic background of the study participants, lack of system training, and low educational level [7, 17, 18]. This review also showed that younger persons were more likely to accept and use e-health systems than people in older age groups. This finding was in line with a systematic study of electronic medical record system adoption in Ethiopia [30, 47]. The possible reason would be the case that younger persons are more willing to adopt new technologies and have a better understanding of ICT than their more experienced peers [30]. This implied that to increase the acceptance of the new health information technology, additional attention should be devoted to older personnel.

## Conclusion

In this study, the best model used to identify facilitators of sustainable acceptance of eHealth in resource-limited settings was the unified theory of acceptance and use of technology, and 12 facilitators and 13 barriers were found to be predictive of Ethiopians’ adoption of the e-health system. The review found that effort expectancy, performance expectancy, facilitating condition, social influence, and attitude were the most frequent predictors of the adoption of e-health systems in Ethiopia. The most common facilitator identified from the predictors was effort expectancy, which played a significant role in the adoption of the e-health system in Ethiopia.

Furthermore, fear of new technology (anxiety) was the frequent barrier to influencing sustainable acceptance of the e-health system in Ethiopia. This insight can help us identify the challenges and potential solutions for Ethiopia’s adoption of an e-health system. Therefore, eHealth implementers and managers in those settings should prioritize improving the technical infrastructure by continuously giving system users basic facilitating conditions; they should also pay attention to the system they want to implement because doing so will improve the system users’ perception of the system’s value and attitude.

### Theoretical implication

Based on this study’s findings. Theoretically, our findings might alleviate any worries about eHealth being accepted in resource-limited settings. Due to the limited evidence on facilitators of sustainable acceptance of e-health. Moreover, the study made numerous contributions to theories and models of technological acceptability, particularly in healthcare. For several reasons, it is thought that this systematic review significantly contributes to the body of literature already in existence. Initially, rather than concentrating on a single model or theory, it examined all technological acceptance models. The acceptance models, extensions, and integrations used in this study were those that had undergone empirical evaluation.

The study looked at several information technologies rather than focusing on just one. The review covered studies with various user groups and contexts. Finally, the reviewed research was published until this study year, giving us a new perspective on the literature.

### Practical implications

The study offers some practical ramifications for the healthcare industry. The inclusion of numerous technology acceptance models, diverse technologies, and diverse consumers sets this study apart from other evaluations in several ways. For other researchers and decision-makers working in various research fields, nations, and situations, this diversity is beneficial. Telemedicine, cloud computing, and mobile applications, for instance, can all greatly enhance the potential of e-health technologies. For doctors and other healthcare workers to deliver numerous healthcare services without physically visiting the patient, decision-makers must give the required support for adopting these solutions, particularly in rural areas.

Practitioners should concentrate on the most significant facilitators of sustainable acceptance of e-health technology for encouraging customers to utilize the product regularly by actively fostering their relationship with them. We think the findings would help policymakers evaluate the regulations and standards currently in existence for data privacy and confidentiality. These regulations should also be declared and made public. To increase their acceptability by raising the degrees of worry and trust, end-users must be informed of their roles and responsibilities.

### Strengths, limitations of the study, and future work

This systematic review contributes to the field by analyzing and summarizing previous studies on e-health acceptance predictors, which may aid in identifying key e-health system implementation determinants. This study has a few significant drawbacks. Despite the authors’ extensive search, only a small number of publications (ten studies) were found. This could be a result of Ethiopia’s inadequate e-health research. Moreover, the study was not included the meta analysis due to the different characteristics of the study and statistics. In the future, this study can be extended to include eHealth solutions and other simulation studies can be conducted in the African context using a different design, population, or setting. Moreover, for enhancing the generalizability of the finding. Future research may extend this review by studying specific e-health systems and include bibliometric analysis to enhance the study findings.

## Supporting information

S1 Table PRISMA checklist to exploring facilitators and barriers of the sustainable acceptance of e-health systems adoption in Ethiopia. (DOCX)

S2 Table Quality assessment of exploring facilitators for the sustainable acceptance of e-health systems adoption in Ethiopia: A systematic review. (DOCX)

S1 Data Dataset. (XLSX)

## Abbreviations

**e-Health**: electronic health
**ICT**: information communication technology
**PRISMA**: Preferred Reporting Items for Systematic Reviews and Meta-Analyses
TAM: Technology Acceptance Model
UTAUT: Unified Theory of Acceptance and Use of Technology

## References

[1] Li J., et al., Health care provider adoption of eHealth: systematic literature review. Interactive journal of medical research, 2013. 2(1): p. e2468. doi: 10.2196/ijmr.2468 23608679 PMC3628149

[2] Rinaldi G., *New Perspectives in Medical Records*: *Meeting the Needs of Patients and Practitioners*. 2017: Springer.

[3] Kalayou M.H., Endehabtu B.F., and Tilahun B., The Applicability of the Modified Technology Acceptance Model (TAM) on the Sustainable Adoption of eHealth Systems in Resource-Limited Settings. J Multidiscip Healthc, 2020. 13: p. 1827–1837. doi: 10.2147/JMDH.S284973 33299320 PMC7721313

[4] Faber S., van Geenhuizen M., and de Reuver M., eHealth adoption factors in medical hospitals: a focus on the Netherlands. International journal of medical informatics, 2017. 100: p. 77–89. doi: 10.1016/j.ijmedinf.2017.01.009 28241940

[5] Dagnew B., et al., Individual and community-level determinants of knowledge of ovulatory cycle among women of childbearing age in Ethiopia: A multilevel analysis based on 2016 Ethiopian Demographic and Health Survey. Plos one, 2021. 16(9): p. e0254094. doi: 10.1371/journal.pone.0254094 34473727 PMC8412270

[6] Atinga R.A., et al., e-health usage and health workers’ motivation and job satisfaction in Ghana. PLoS One, 2020. 15(9): p. e0239454. doi: 10.1371/journal.pone.0239454 32966323 PMC7510985

[7] Walle A.D., et al., Readiness to use electronic medical record systems and its associated factors among health care professionals in Ethiopia: A systematic review and meta-analysis. Informatics in Medicine Unlocked, 2022: p. 101140.

[8] Wilson J., et al., Barriers and facilitators to the use of e-health by older adults: a scoping review. BMC Public Health, 2021. 21(1): p. 1556. doi: 10.1186/s12889-021-11623-w 34399716 PMC8369710

[9] Melchiorre M.G., et al., eHealth in integrated care programs for people with multimorbidity in Europe: Insights from the ICARE4EU project. Health policy, 2018. 122(1): p. 53–63. doi: 10.1016/j.healthpol.2017.08.006 28899575

[10] Torrent-Sellens J., et al., Modelling and predicting eHealth usage in Europe: a multidimensional approach from an online survey of 13,000 european union internet users. Journal of medical Internet research, 2016. 18(7): p. e5605. doi: 10.2196/jmir.5605 27450189 PMC4975796

[11] van Hilten O., Gezondheid en zorg in cijfers 2009. TSG, 2010. 88(2): p. 63–63.

[12] Van Ewijk, C., A. Van der Horst, and P. Besseling, *The future of health care*. 2013.

[13] Nijland, N., *Grounding eHealth*: *towards a holistic framework for sustainable eHealth technologies*. 2011.

[14] Lee J., et al., The adoption gap: Health information technology in small physician practices. Health Affairs, 2005. 24(5): p. 1364–1366. 16162585 10.1377/hlthaff.24.5.1364

[15] Fanta, G.B., L. Pretorius, and L. Erasmus, *Organizational Dynamics of Sustainable eHealth Implementation*: *A Case Study of eHMIS*. 2017 Portland International Conference on Management of Engineering and Technology (PICMET), 2017: p. 1–9.

[16] Vishwanath A. and Scamurra S.D., Barriers to the adoption of electronic health records: using concept mapping to develop a comprehensive empirical model. Health Informatics J, 2007. 13(2): p. 119–34. doi: 10.1177/1460458207076468 17510224

[17] Walle A.D., et al., Predicting healthcare professionals’ acceptance towards electronic personal health record systems in a resource-limited setting: using modified technology acceptance model. BMJ Health & Care Informatics, 2023. 30(1): p. e100707. 10.1136/bmjhci-2022-100707 PMC9990677 36878620

[18] Walle A.D., et al., Intention to use wearable health devices and its predictors among diabetes mellitus patients in Amhara region referral hospitals, Ethiopia: Using modified UTAUT-2 model. Informatics in Medicine Unlocked, 2023. 36: p. 101157.

[19] Gashu K.D., et al., Does phone messaging improves tuberculosis treatment success? A systematic review and meta-analysis. BMC Infectious Diseases, 2020. 20(1): p. 42. doi: 10.1186/s12879-020-4765-x 31937260 PMC6961375

[20] Tilahun B. and Fritz F., Modeling antecedents of electronic medical record system implementation success in low-resource setting hospitals. BMC Medical Informatics and Decision Making, 2015. 15(1): p. 61. doi: 10.1186/s12911-015-0192-0 26231051 PMC4522063

[21] Mekonnen Z.A., et al., Mothers intention and preference to use mobile phone text message reminders for child vaccination in Northwest Ethiopia. BMJ Health Care Inform, 2021. 28(1). doi: 10.1136/bmjhci-2020-100193 33608258 PMC7898827

[22] Al-Emran M. and Arpaci I., Intelligent systems and novel coronavirus (Covid-19): A bibliometric analysis. Emerging technologies during the era of COVID-19 pandemic, 2021: p. 59–67.

[23] Rahimi B., et al., A systematic review of the technology acceptance model in health informatics. Applied clinical informatics, 2018. 9(03): p. 604–634. doi: 10.1055/s-0038-1668091 30112741 PMC6094026

[24] AlQudah A.A., Al-Emran M., and Shaalan K., Technology acceptance in healthcare: A systematic review. Applied Sciences, 2021. 11(22): p. 10537.

[25] AlQudah A.A., et al., Toward an Integrated Model for Examining the Factors Affecting the Acceptance of Queue Management Solutions in Healthcare. IEEE Transactions on Engineering Management, 2022.

[26] Venkatesh V., et al., User acceptance of information technology: Toward a unified view. MIS quarterly, 2003: p. 425–478.

[27] Kim S., et al., Analysis of the factors influencing healthcare professionals’ adoption of mobile electronic medical record (EMR) using the unified theory of acceptance and use of technology (UTAUT) in a tertiary hospital. BMC medical informatics and decision making, 2015. 16(1): p. 1–12. 10.1186/s12911-016-0249-8 PMC4736616 26831123

[28] Goodarzian F., et al., Designing a green home healthcare network using grey flexible linear programming: Heuristic approaches. Journal of Computational Design and Engineering, 2021. 8(6): p. 1468–1498.

[29] Tegegne M.D., et al., Health information seeking and its associated factors in Ethiopia: Systematic review and meta-analysis. Informatics in Medicine Unlocked, 2022: p. 100980.

[30] Tegegne M.D., et al., Electronic Medical Record System Use and Determinants in Ethiopia: Systematic Review and Meta-Analysis. Interactive Journal of Medical Research, 2023. 12(1): p. e40721. doi: 10.2196/40721 36630161 PMC9878362

[31] Ahmed M.H., et al., Intention to use electronic medical record and its predictors among health care providers at referral hospitals, north-West Ethiopia, 2019: using unified theory of acceptance and use technology 2 (UTAUT2) model. BMC Medical Informatics and Decision Making, 2020. 20(1): p. 1–11. 32883267 10.1186/s12911-020-01222-x PMC7469309

[32] Bramo S.S., Desta A., and Syedda M., Acceptance of information communication technology-based health information services: Exploring the culture in primary-level health care of South Ethiopia, using Utaut Model, Ethnographic Study. Digital Health, 2022. 8: p. 20552076221131144. doi: 10.1177/20552076221131144 36276184 PMC9585563

[33] Hunde M.K., Demsash A.W., and Walle A.D., Behavioral intention to use e-learning and its associated factors among health science students in Mettu university, southwest Ethiopia: Using modified UTAUT model. Informatics in Medicine Unlocked, 2022: p. 101154.

[34] Shiferaw K.B. and Mehari E.A., Modeling predictors of acceptance and use of electronic medical record system in a resource limited setting: Using modified UTAUT model. Informatics in Medicine Unlocked, 2019. 17: p. 100182.

[35] Shiferaw K.B., et al., Healthcare providers’ acceptance of telemedicine and preference of modalities during COVID-19 pandemics in a low-resource setting: An extended UTAUT model. Plos one, 2021. 16(4): p. e0250220. doi: 10.1371/journal.pone.0250220 33886625 PMC8061916

[36] Kifle M., et al., Transfer and adoption of advanced information technology solutions in resource-poor environments: the case of telemedicine systems adoption in Ethiopia. Telemedicine and e-Health, 2010. 16(3): p. 327–343. doi: 10.1089/tmj.2009.0008 20406120

[37] Rouidi M., et al., TAM-UTAUT and the acceptance of remote healthcare technologies by healthcare professionals: A systematic review. Informatics in Medicine Unlocked, 2022: p. 101008.

[38] Gu D., et al., Assessing the adoption of e-health technology in a developing country: an extension of the UTAUT model. Sage Open, 2021. 11(3): p. 21582440211027565.

[39] Semwanga, A.R., et al., *An ehealth Adoption Framework for Developing Countries*: *A Systematic Review*. 2021.

[40] Peek S.T., et al., Factors influencing acceptance of technology for aging in place: a systematic review. International journal of medical informatics, 2014. 83(4): p. 235–248. doi: 10.1016/j.ijmedinf.2014.01.004 24529817

[41] Garavand A., et al., Factors influencing the adoption of health information technologies: a systematic review. Electronic physician, 2016. 8(8): p. 2713. doi: 10.19082/2713 27757179 PMC5053450

[42] Binyamin S.S. and Zafar B.A., Proposing a mobile apps acceptance model for users in the health area: A systematic literature review and meta-analysis. Health Informatics Journal, 2021. 27(1): p. 1460458220976737. doi: 10.1177/1460458220976737 33438494

[43] Garavand A., et al., Acceptance of telemedicine technology among physicians: A systematic review. Informatics in Medicine Unlocked, 2022: p. 100943.

[44] Khalil, A.-A., A.N. Hidayanto, and H. Prabowo. *Identification of factor Affecting continuance usage intention of mHealth application*: *a systematic literature review*. *in 2020 4th International Conference on Informatics and Computational Sciences (ICICoS)*. 2020. IEEE.

[45] Tao, D., et al. *Predicting factors of consumer acceptance of health information technologies*: *a systematic review*. in *Proceedings of the Human Factors and Ergonomics Society Annual Meeting*. 2016. SAGE Publications Sage CA: Los Angeles, CA.

[46] Wang T., et al., Identifying major impact factors affecting the continuance intention of mHealth: a systematic review and multi-subgroup meta-analysis. npj Digital Medicine, 2022. 5(1): p. 145. doi: 10.1038/s41746-022-00692-9 36109594 PMC9476418

[47] Yehualashet D.E., et al., Barriers to the adoption of electronic medical record system in Ethiopia: a systematic review. Journal of Multidisciplinary Healthcare, 2021: p. 2597–2603. doi: 10.2147/JMDH.S327539 34556994 PMC8455291

