# Supplementary material for: Exploring facilitators and barriers of the sustainable acceptance of e-health system solutions in Ethiopia: A systematic review
Source: PLoS One. 2023 Aug 10;18(8):e0287991. doi: 10.1371/journal.pone.0287991 (PMC10414679; doi:10.1371/journal.pone.0287991)
Supplement: S2 Table — (DOCX) [file pone.0287991.s002.docx]

S2 Table : Quality assessment of exploring facilitators for the sustainable acceptance of e-health systems adoption in Ethiopia: A systematic review

| Author, year of  Study | Q1 | Q2 | Q3 | Q4 | Q5 | Q6 | Q7 | Q8 | Q9 | Total score (9%) |
| --- | --- | --- | --- | --- | --- | --- | --- | --- | --- | --- |
| Ahmed. et al,2020 | Y | Y | Y | Y | Y | Y | NA | Y | Y | 8 |
| Shiferaw. et al, 2019 | Y | Y | Y | Y | Y | NA | Y | NA | Y | 7 |
| Kalayou. et al, 2020 | Y | Y | Y | Y | Y | Y | Y | Y | Y | 9 |
| Walle. et al, 2022 | Y | Y | Y | Y | Y | Y | Y | Y | Y | 9 |
| Shiferaw. et al, 2021 | Y | Y | Y | NA | Y | Y | Y | Y | Y | 8 |
| Mekonnen. et al, 2021 | Y | Y | Y | Y | Y | Y | Y | Y | Y | 9 |
| Hunde. et al,2022 | Y | Y | Y | Y | Y | Y | Y | Y | Y | 9 |
| kiflie.et al,2010 | Y | Y | Y | Y | Y | Y | NA | NA | Y | 7 |
| Bramo.et al, 2022 | Y | Y | Y | Y | Y | Y | Y | Y | Y | 9 |
| Walle.et.al 2023 | Y | Y | Y | Y | Y | Y | Y | Y | Y | 9 |

**Key:** **Y**= Yes; **NR**= Not reported, **NA**=Not appropriate

**Question codes:**

1. Was the sample frame appropriate to address the target population?

2. Were study participants sampled in an appropriate way?

3. Was the sample size adequate?

4. Were the study subjects and the setting described in detail?

5. Was the data analysis conducted with sufficient coverage of the identified sample?

6. Were valid methods used for the identification of the condition?

7. Was the condition measured in a standard, reliable way for all participants?

8. Was there appropriate statistical analysis?

9. was the response rate adequate, and if not, was the low response rate managed appropriately?
